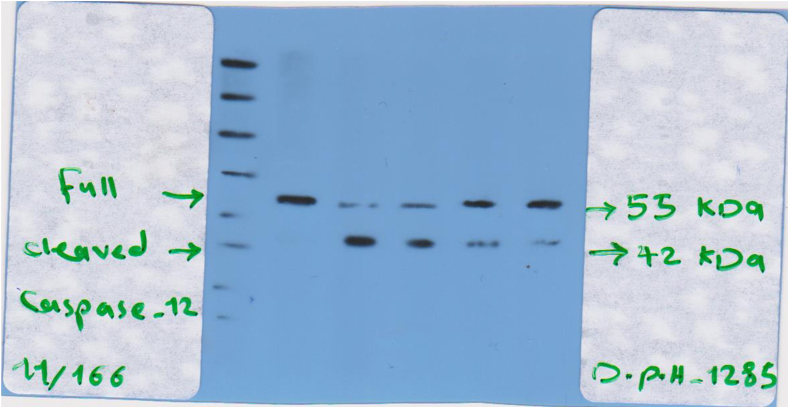# Endoplasmic reticulum stress PERK-ATF4-CHOP pathway is involved in non-alcoholic fatty liver disease in type 1 diabetic rats: The rescue effect of treatment exercise and insulin-like growth factor I

**DOI:** 10.1016/j.heliyon.2024.e27225

**Published:** 2024-03-01

**Authors:** Shadi Mohammadpour-Asl, Behrad Roshan-Milani, Shiva Roshan-Milani, Ehsan Saboory, Bijan Ghobadian, Leila Chodari

**Affiliations:** a Student Research Committee, Urmia University of Medical Sciences, Urmia, Iran; b Department of Physiology, School of Medicine, Urmia University of Medical Sciences, Urmia, Iran; c Neurophysiology Research Center, Cellular and Molecular Medicine Research Institute, Urmia University of Medical Sciences, Urmia, Iran; d Department of Addiction Studies, School of Medicine, Zanjan University of Medical Sciences, Zanjan, Iran; e Zanjan Metabolic Diseases Research Center, Zanjan University of Medical Sciences, Zanjan, Iran

**Keywords:** Diabetes, Non-alcoholic fatty liver disease, Endoplasmic reticulum stress, Exercise, Insulin-like growth factor I

## Abstract

Endoplasmic Reticulum Stress (ERS) is a key factor in the development of Non-Alcoholic Fatty Liver Disease (NAFLD) in diabetes. The current study aimed to examine the effects of exercise and IGF-I on ERS markers in liver tissue. Rats were divided into five groups (n = 8 per group), including control (CON), diabetes (DIA), diabetes + exercise (DIA + EX), diabetes + IGF-I (DIA + IGF-I), and diabetes + exercise + IGF-I (DIA + EX + IGF-I). Type 1 diabetes was induced by an I.P. injection of streptozotocin (60 mg/kg). After 30 days of treatment with exercise or IGF-I alone or in combination, liver tissue was assessed for caspase 12, 8, and CHOP protein levels, and expression of ERS markers (ATF-6, PERK, IRE-1A) and lipid metabolism-involved genes (FAS, FXR, SREBP-1c) by western immunoblotting. In addition, for the evaluation of histopathological changes in the liver, Hematoxylin - Eosin and Masson's Trichrome staining were done. Compared to the control group, diabetes significantly caused liver fibrosis, induced ERS, increased caspase 12 and 8 levels in the liver, and changed expression levels of genes associated with lipid metabolism, including FAS, FXR, and SREBP-1c. Treatment with either exercise or IGF-I reduced fibrosis levels suppressed ER stress markers and apoptosis, and improved expression of genes associated with lipid metabolism. In addition, simultaneous treatment with exercise and IGF-I showed a synergistic effect compared to DIA + E and DIA + IGF-I. The results suggest that IGF-1 and exercise reduced liver fibrosis possibly by reducing ERS, creating adaptive ER stress status, and improving protein folding.

## 1 Introduction

Non-alcoholic fatty liver disease (NAFLD) is a widespread public health problem that is thought to be the primary cause of liver diseases linked to hyperlipidemia, metabolic syndrome, diabetes, obesity, and other risk factors [1,2]. NAFLD is known to frequently occur in people with diabetes mellitus (DM), which is characterized by abnormal lipid metabolism and excess triglycerides (TGs) accumulated in lipid droplets [3,4]. Increased levels of free fatty acids (FFAs) in the liver of diabetic individuals promote the development of steatohepatitis, a condition characterized by steatosis, inflammation, apoptosis, and fibrosis, as well as end-stage liver disease [5]. Although the link between NAFLD and DM is the subject of extensive research, the exact molecular pathways have not yet been determined [6,7].

Endoplasmic reticulum (ER) stress has been shown to play a significant role in hepatic lipid metabolism and cellular damage, suggesting a potential mechanistic role in the link between diabetes and NAFLD [8,9]. The accumulation of misfolded/unfolded proteins within the ER induces ER stress and under ER stress conditions, a series of conserved signaling pathways named the unfolded protein response (UPR), are activated to restore normal ER function [10]. UPR pathways result in either “adaptive UPR,” in which ER homeostasis is provided, or “maladaptive UPR,” in which the apoptotic process is designed and is a consequence of prolonged and sustained activation of the UPR [11]. Three UPR signaling pathways are protein kinase RNA-like ER kinase (PERK), inositol requiring protein 1 (IRE-1), and activating transcription factor 6 (ATF6), which are ER-resident proteins known to recognize ER stress in cells [12]. Under ER stress conditions, GRP78/BiP, a chaperone in the ER membrane, separates from the membrane, recognizes misfolded/unfolded proteins, and induces three UPR pathways to restore ER homeostasis [13].

Several studies have shown that ERS affects the expression of several genes involved in lipid metabolism, can regulate several aspects of cellular metabolism, and even affect the cell's fate [14,15]. So, managing the ERS pathway may be a successful tactic for reducing the degree and severity of NAFLD.

SREBP-1c controls the expression of de novo lipogenesis-related genes such as Fans, SCD-1, and Acyl, which primarily regulate hepatic lipogenesis [16]. Hepatic steatosis has been linked to increased SREBP-1c expression in multiple cases [17]. ATF-6, one of the ER stress proximal sensors, and SREBP-1 are both activated by the same proteases, hypothesizing that ER stress and SREBP activation are related. It has been demonstrated that ER stress, irrespective of its cause (disturbance of calcium homeostasis, glycosylation, or redox status), promotes fast cleavage of SREBP-1c precursor form and production of SREBP-1c target genes without requiring insulin [64].

On the other hand, the farnesoid X receptor (FXR) oppositely regulates SREBP-1c and strictly controls the expression of SREBP-1c [16]. Hyperlipidemia and hepatic steatosis were seen in FXR-deficient mice [18]. It is reported that NAFLD patients had significantly lower levels of hepatic FXR expression, which suggests that FXR failure may be a contributing factor in the onset of NAFLD [19]. Prior studies demonstrated that endoplasmic reticulum stress-mediated downregulation of FXR in aging mice exacerbates hepatic steatosis [20].

A multi-enzyme protein called fatty acid synthase (FAS) encourages lipid production processes in the liver. Liver expression levels of FAS in diabetes increase, which will lead to fat accumulation and induce NAFLD [21].

Recently, it has been demonstrated that physical activity, such as treadmill exercise, improves NAFLD in diabetic models. Also, exercise has been shown to decrease endoplasmic reticulum stress and associated signaling pathways in the cardiac tissue of diabetic rats.

IGF-I, a peptide hormone with structural similarities to insulin, has a substantial proliferative and anti-apoptotic effect on target tissues. Additionally, it has been demonstrated that administering IGF-I to adults with type 1 diabetes improves insulin sensitivity by elevating systemic IGF-I levels.

Based on previous studies, we hypothesized that exercise alone or in combination with IGF-I could reduce/suppress ER stress and, as a result, decrease NAFLD markers in diabetic rats. For this purpose, we evaluated the gene expression of possible molecules involved in the ER stress signaling pathways (PERK-ATF4-IRE-1) and NAFLD (FXR, SREBP-1c, FAS).

## 2 Materials and methods

### 2.1 Ethical approval

Animal Care and experimental techniques were conducted according to the US National Institutes of Health's Guide for the Care and Use of Laboratory Animals (NIH publication no. 85–23, revised 2011), and the journal's ethical values and our study complies with this animal ethics checklist. The ethical research code was authorized by Urmia University of Medical Sciences' animal ethics committee, 1399.099). We have taken all necessary steps to alleviate the pain and suffering of the animals. At the end of the study, the animals were anesthetized using a combination of 100 mg/kg of 10% ketamine and 5 mg/kg of 2% xylazine, administered into the intraperitoneal region.

### 2.2 Animals and study design

Forty male Wistar rats were purchased from Urmia University of Medical Sciences' animal housing. The animals had free access to water and food and were kept in standard conditions with lights on from 8:00 to 20:00 at a temperature of 22 °C. A total of 40 male Wistar rats, weighing 250 ± 10 g, were chosen at random to form the following five groups (n = 8): control (CON), diabetes (DIA), diabetes + exercise (DIA + EX), diabetes + IGF-I (DIA + IGF-I), and diabetes + exercise + IGF-I (DIA + EX + IGF-I). Five rats at most were kept in each cage. Interventions were carried out for one month. Fig. 1 illustrates the design of the study.

**Fig. 1:**
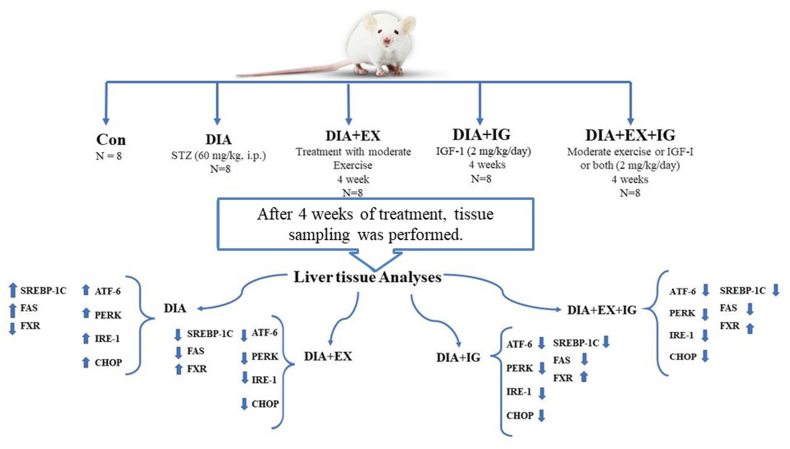
Schematic image of study design.

### 2.3 Induction of diabetes

Streptozotocin (Sigma, USA) was injected intraperitoneally (IP) into the rats once to cause type 1 diabetes. To prepare the IP solution, streptozotocin was dissolved in a solution containing 10 mM sodium citrate and 0.9 percent NaCl, with a pH of 4.5. After 72 h, blood glucose concentrations were evaluated using a glucometer, which confirmed the presence of diabetes. Diabetes was diagnosed in rats at blood glucose levels of 300 mg/dl.

### 2.4 Exercise protocol

Rats in the exercise group were first acclimated to running on a treadmill for one week; and then they exercised for four weeks with a training schedule that included running on a treadmill at a speed of 17 m/min without the tendency for 10 min on the first day, with slight increases of 5 min every day (+5 min), reaching 30 min on the fifth day, which was then preserved for the following three weeks. This exercise program is considered to provide moderate activity [22]. Rats that were not exercised were handled the same way as the exercised rats. All trials took place between 9:00 and 12:00 h.

### 2.5 IGF-I treatment

For four weeks, the rats received 2 mg/kg/day of IGF-I (Sigma Aldrich, USA). In the control group, rats received 0.2 mL of standard saline injections [23].

### 2.6 Tissue processing and measurement of proteins

After the experiment, xylazine (5 mg/kg) and ketamine (80 mg/kg) intraperitoneal injections were administered to all animals to induce unconsciousness. The liver was then removed and instantly frozen using liquid nitrogen. Liver tissue samples were utilized to evaluate protein levels by Western Blotting assay and were kept at 70 °C until ATF-6, PERK, IRE-1A, Caspase 12 and 8, CHOP, SREBP-1c, FAXR, and FAS were measured. After homogenizing the liver samples in RIPA buffer (pH 7.2–7.3) at 4 °C and 1000 g, the samples were centrifuged for 20 min. Target proteins were isolated from the resultant supernatants and quantified using the manufacturer's recommendations for ATF-6, PERK, IRE-1A, Caspase 12, Caspase 8, CHOP, SREBP-1c, FAXR, and FAS.

### 2.7 Western blotting procedure

The expression of ATF-6, PERK, IRE-1A, Caspase 12, Caspase 8, CHOP, SREBP-1c, FXR, and FAS in liver tissue was evaluated by western immunoblotting. The Bradford assay kit was used to quickly measure the concentration of the supernatant (Sigma Aldrich, USA). After mixing a 20 g protein and a 2X sample loading buffer, each well was loaded. All the loaded samples' proteins were separated in 10% SDS-gels and transferred to PVDF membranes within an hour. The membranes were first blocked for 1.5 h in a solution containing 5% skim milk and 0.1% Tween-20. The membranes were then probed overnight at 4 °C in a shaker incubator with primary antibodies against ATF-6 (SAB2100170), PERK (P0074 Anti-PEK), IRE-1A (ZRB1072-4X25UL), Caspase 12 (MFCD03454855), Caspase 8 (C4106 Anti Cas 8), CHOP (SAB4500631), SREBP-1c (SAB1412393), FXR (SAB2500434), and FAS (AB16982).

A secondary HRP-conjugated antibody was applied to the membranes (1:7000, cell signaling) for 4–5 min after being washed with a Tris-buffered saline solution containing 0.1% Tween-20. After 1 h of shaking incubation, the membranes were immersed in the wash buffer and washed for at least three to 5 min. The membranes were then exposed to enhanced chemiluminescence (ECL, Amersham) reagents in a darkened room. The binding's chemiluminescence was then seen utilizing a visualizing device after the membrane had been exposed to an X-ray beam. The intensity of the bands was determined using Image J software and normalized to the bars of the internal control (beta-actin).

### 2.8 Biochemistry analysis

Colorimetric and enzymatic methods were used to assess total plasma cholesterol (TC) and triglycerides (TGs). The manufacturer's instructions were followed to assess the plasma levels of liver enzymes such as alanine aminotransferase (ALT), aspartate aminotransferase (AST), and alkaline phosphatase (ALP) using the colorimetric assay.

### 2.9 Histopathological examinations (hematoxylin - Eosin and Masson's trichrome staining)

To assess overall histological alterations and steatosis, Hematoxylin and Eosin (H&E) staining was used. To preserve the liver tissue, it was fixed in 10% neutral buffered formalin (37%, Merck) until it could be used. After being dehydrated in a series of decreasing concentrations of ethanol, the tissues were cleared in xylene and embedded in paraffin. The sections were cut to a 4-μm thickness and deparaffinized with xylene. The tissue section was rehydrated using ethanol solutions of increasing concentrations. To prepare for staining, the sections were washed with sterile distilled water. As described elsewhere [24], sections were stained with hematoxylin,and counterstained with eosin, and mounted in DPX for hematoxylin and eosin staining. The images were taken using an Olympus IX camera.

To assess liver tissue fibrosis, tissue sections were stained using a Masson's Trichrome kit (Betagen, Tehran, Iran), following the manufacturer's instructions.

As previously reported [25], liver sections that had been rehydrated and washed were stained with Masson's trichrome. A phosphomolybdic-phosphotungstic acid solution was used to differentiate Biebrich scarlet-acid fuchsin solution. After staining with aniline blue solution, dissecting in 1% acetic acid, and counterstaining with Weigert iron hematoxylin, the sections were counterstained with Weigert iron hematoxylin. To dehydrate the slides, ascending concentrations of ethanol were used, followed by clearing in xylene and mounting in DPX (Sigma-Aldrich). Olympus IX81 inverted light microscope was used to visualize sections. A score ranging from zero (normal liver) to four (severe fibrosis) was used. The grades appointed in scoring liver fibrosis were as follows: Grade 0: Normal liver; Grade 1: Minimal fibrosis thickening of liver tissue; Grade 2: Moderate thickening of liver tissue without obvious damage to the structure of liver tissue; Grade 3: Increased fibrosis with definite damage to the architecture of the liver and formation of fibrosis bands or small fibrosis masses; Grade 4: Severe distortion of the structure and large fibrosis areas.

### 2.10 Statistical analysis

The results are expressed as mean ± SEM. SPSS 18 was used to perform statistical analyses at a significance level of P < 0.05. The Kolmogorov-Smirnov test revealed that all the data had a normal distribution. Therefore, normally distributed data were analyzed using one-way parametric analysis of variance (ANOVA), followed by the LSD post hoc test. A p-value of less than 0.05 was regarded as statistically significant.

## 3 Results

### 3.1 Effects of IGF-I and exercise on endoplasmic reticulum stress protein (IRE-1, ATF-6, PERK, and CHOP) levels in the liver tissue

The expression levels of endoplasmic reticulum stress proteins (IRE-1, ATF-6, PERK, and CHOP) were investigated by western blotting (Fig. 2A–E). According to Fig. 2A, the IRE-1 protein levels of liver tissue significantly increased in the diabetic group compared to the control group (P < 0.05). Additionally, the 4-week treatment of the diabetic rats with exercise showed a significant decrease in IRE-1 protein levels compared to the DIA group (Fig. 2A,P < 0.001). Furthermore, increased levels of IRE-1 protein as a result of treatment with IGF-I compared to the DIA group were observed. Treatment with IGF-I led to a decrease in IRE-1 protein levels in the liver tissue (P < 0.001). Interestingly, the IRE-1 level was considerably reduced in the IGF-I and exercise combination treatment group compared to the DIA, DIA + EX, and DIA + IGF-I groups (P < 0.001). Fig. 2B and (C) show that protein levels of PERK and ATF-6 in the liver of the DIA group appeared to be significantly higher than those in the control rats (P < 0.001). Moreover, exercise treatment in the DIA + EX group significantly decreased PERK (Fig. 2B, P < 0.001) and ATF-6 (Fig. 2C, P < 0.05) protein levels compared with the DIA group. However, exercise treatment had a greater effect on PERK (P < 0.001) and a lesser effect on ATF-6 protein levels than the DIA group. According to the figure, rats in the DIA + IGF-I group had a considerably lower PERK protein level than those in the DIA group (P < 0.001). It was found that simultaneous treatment with exercise and IGF-I decreased the PERK protein level significantly compared with the DIA, DIA + EX, and DIA + IGF-I groups (P < 0.001).

**Fig. 2:**
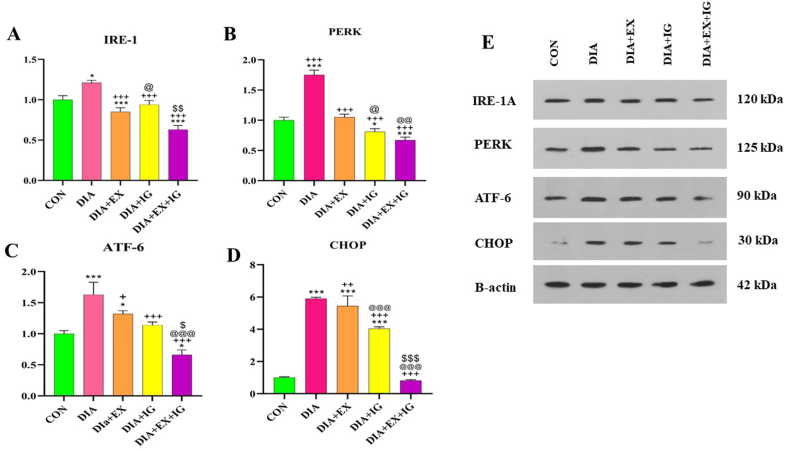
Effect of IGF-I and exercise on endoplasmic reticulum protein levels (IRE-1, PERK, ATF-6, and CHOP) in diabetic and healthy rats. (E) Immunoblotting images of expression level of (IRE-1, PERK, ATF-6, and CHOP). (A–D) Bar graphs represent the relative density of each band normalized to that of β-actin as an internal control. Bars represent the Mean ± SEM (*n* = 8). *P < 0.05 and ***P < 0.001 *versus* the control (CON) group. +P < 0.05, ++P < 0.01, ++P < 0.001 *versus* the diabetes (DIA) group. @ P < 0.05, @@ P < 0.01, @@@ P < 0.001 versus the exercise (DIA + EX) group. $ P < 0.05, $$ P < 0.01, $$$ P < 0.001 versus the IGF-I (DIA + IG) group.

Moreover, the ATF-6 protein level in the DIA + EX + IGF-I group was significantly lower than that in the DIA (P < 0.001), DIA + EX, and DIA + IGF-I (Fig. 2C,P < 0.05) groups.

In addition, a significant (P < 0.001) increase in levels of CHOP protein as a result of diabetes compared to the control group was observed. In comparison to the DIA group, the DIA + EX group and the DIA + IGF-I group exhibited a significant reduction in CHOP protein level (Fig. 2D,P < 0.01, P < 0.001 respectively).

The results show that co-treatment of exercise and IGF-I in the DIA + EX + IGF-I group hassignificantly decreased CHOP protein level compared with the DIA group and it approximately reached the level of the control group (P < 0.001).

### 3.2 Effects of IGF-I and exercise on lipid metabolism involved genes SREBP-1C, FAS, and FXR protein levels

According to Fig. 3A, the SREBP-1C levels of liver tissue significantly (P < 0.001) increased in the diabetic group compared to the control group. When diabetic rats were treated with exercise and IGF-I, the SREBP-1C protein level significantly decreased compared to the DIA rats (P < 0.001). Also, the SREBP-1C level in the liver tissue of the DIA + EX + IGF-I group was significantly lower (P < 0.001) than that of the DIA group.

**Fig. 3:**
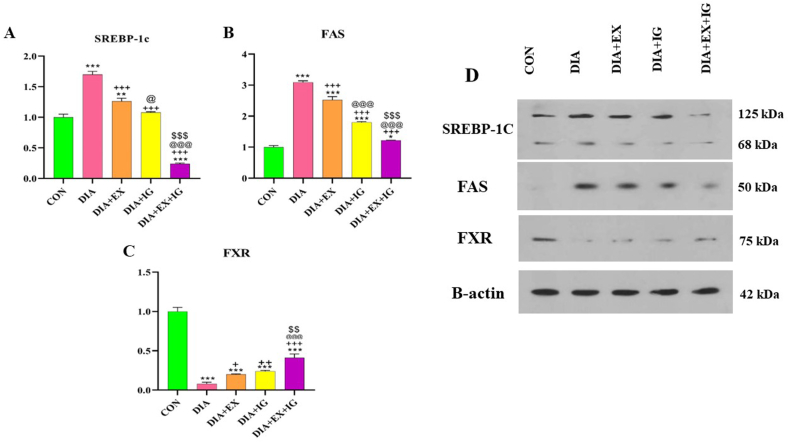
Effect of IGF-I and exercise on endoplasmic reticulum protein levels (SREBP-1C, FAS, and FXR) in diabetic and healthy rats. (D) Immunoblotting images of expression of SREBP-1C, FAS, and FXR. Quantitative densitometric analysis of SREBP-1C, FAS, and FXR protein bands (A–C). The values are shown as mean ± SEM (n = 8); *P < 0.05 and ***P < 0.001 versus the control (CON) group. ++P < 0.05, ++P < 0.01, ++P < 0.001 versus the diabetes (DIA) group. @ P < 0.05, @@@ P < 0.001 versus the exercise (DIA + EX) group. $$ P < 0.01, $$$ P < 0.001 versus the IGF-I (DIA + IG) group.

Fig. 3B shows a significant (P < 0.001) increase in FAS protein level in the diabetic group compared to the control group. FAS protein level was significantly lower in the IGF-I-treated group than in the DIA treatment group (P < 0.001). A statistical analysis of combined therapy demonstrated that DIA + IGF-I + EX significantly decreased (P < 0.001) FAS protein levels, compared to the DIA, DIA + IGF-I, and DIA + EX groups.

According to the results, reduced levels of FXR protein were detected in the liver of diabetic rats compared to the control group (P < 0.001). According to Fig. 3C, treatment with IGF-I (P < 0.01) and exercise for one month showed a significant (P < 0.05) increase in FXR protein level in the DIA + IGF-I and DIA + EX groups compared to the DIA group. Moreover, the exercise and IGF-I co-administration significantly decreased FXR protein levels in comparison to the DIA group (Fig. 3C, P < 0.001).

### 3.3 Effects of IGF-I and exercise on plasma lipid profile, and liver enzyme levels

Following a four-week course of treatment, Fig. 4A exhibits changes in the liver enzyme profile and plasma lipid profile in various experimental groups. A higher level of cholesterol in plasma was noted in the diabetic rat group when compared to the control group (P < 0.001). The plasma cholesterol level of the exercise group (P < 0.01) and the IGF-I group (P < 0.001) was significantly lower than that of the DIA group. Fig. 4A also shows a decrease in cholesterol levels associated with simultaneous treatment with IGF-I and exercise as compared to the DIA (P < 0.001).

**Fig. 4:**
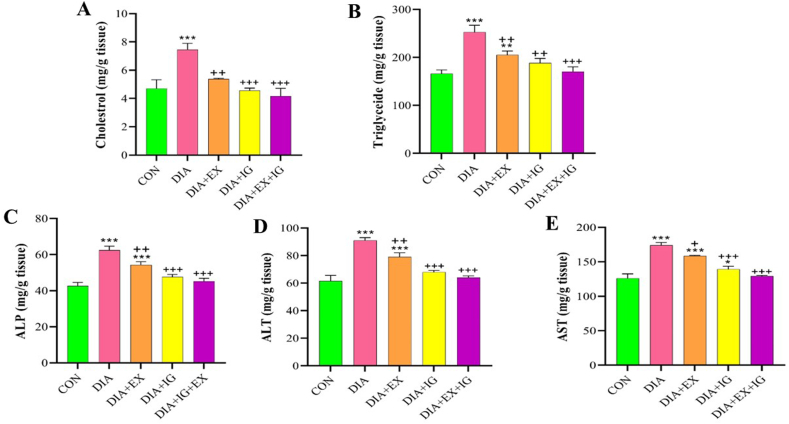
Effect of IGF-I and exercise on Plasma lipid profile (A,B), and liver enzyme levels (C–E) in diabetic and healthy rats. Bars represent the Mean ± SEM (*n* = 8). *P < 0.05 and ***P < 0.001 *versus* the control (CON) group. ++P < 0.05, ++P < 0.01, ++P < 0.001 *versus* the diabetes (DIA) group.

A similar trend is seen in the DIA group compared with the control group in terms of triglyceride levels (Fig. 4B). A significant (P < 0.01) decrease in triglyceride level was observed in the DIA + IGF-I and DIA + EX groups compared with the DIA group. Further, the co-treatment of IGF-I with exercise significantly reduced triglyceride levels compared to the DIA (P < 0.001) groups (Fig. 4B). Regarding the liver enzymes, the diabetes condition significantly (P < 0.001) increased ALP, ALT, and AST levels in comparison with the control group (Fig. 4C–E).

Additionally, exercise treatment and IGF-I administration were significantly associated with lower liver ALP, ALT, and AST levels compared to the DIA group. The result showed that co-treatment with IGF-I and exercise significantly reduced the level of ALT and AST in the liver compared to the DIA + IGF (P < 0.001 for both) and DIA + EX (P < 0.01, P < 0.05 respectively) groups (Fig. 4D and E).

### 3.4 Effects of exercise and IGF-I on caspase 8 and 12 protein levels

One key factor causing caspase activation could be ER Stress. In accordance, the protein expression levels of caspase-8 and caspase-12 (as apoptotic death markers) were investigated by western blotting. As shown in Fig. 5A,B, both c-caspase8/t-caspase8 and c-caspase12/t-caspase12 ratios significantly (P < 0.001) increased in the DIA group compared to the control group. Both exercise and IGF-I treatment markedly reversed the liver tissue's DIA-induced enhancement of the c-caspase 8/t-caspase 8 and c-caspase12/t-caspase12 ratios (P < 0.001).

**Fig. 5:**
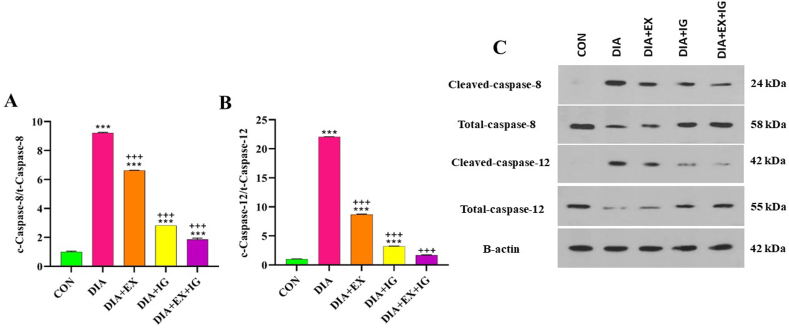
Effect of IGF-I and exercise on Caspase 8 and 12 protein levels in diabetic and healthy rats. (C) Immunoblotting images of expression of caspase8 (cleaved caspase 8/total-caspase8 ratio) and caspase12 (cleaved caspase 12/total-caspase12 ratio). (A,B). Quantitative densitometric analysis of SREBP-1C, FAS, and FXR protein bands. The values are shown as mean ± SEM (n = 8); *P < 0.05 and ***P < 0.001 *versus* the control (CON) group. ++P < 0.05, ++P < 0.01, ++P < 0.001 *versus* the diabetes (DIA) group. @@@ P < 0.001 versus the exercise (DIA + EX) group. $$$ P < 0.001 versus the IGF-I (DIA + IG) group.

Interestingly, a significant reduction of caspase-8 and caspase-12 was observed in the DIA + EX + IGF-I group when compared with the DIA + IGF-I and DIA + EX (P < 0.001). Results for the activity of caspase-8 and 12 are summarized in Fig. (5).

### 3.5 Effects of exercise and IGF-I on histopathological changes

According to Fig. 6(A-E, a-e) H&E staining results showed that diabetes significantly causes portal lymphocytic inflammation, high levels of lipid droplets, vacuolar degeneration of hepatocytes edema, hyperemia, and hepatocellular ballooning compared with the control group. Conversely, treatment with both exercise and IGF-I reduced the levels of liver cell damage followed by low to moderate inflammation compared to the diabetes group. Also, a significant reduction of liver injuries was observed in the DIA + EX + IGF-I groups when compared with the DIA + IGF-I and DIA + EX groups.

**Fig. 6:**
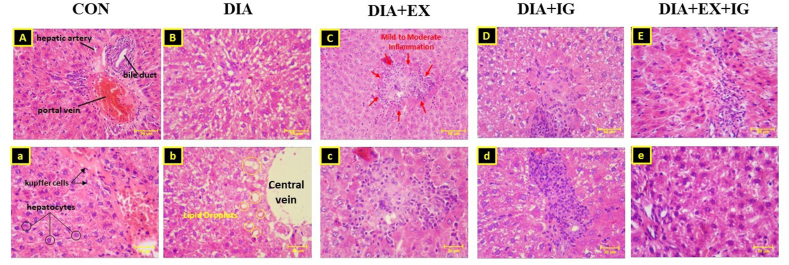
(A-E, a-e): photomicrographs of hematoxylin and eosin-stained sections of liver tissues from each group are shown with 200 (A–E) and 400 (a–e) magnification.

According to Fig. 7(A-E), Masson's trichrome staining, there were no lesion scores in the liver tissue of the control group (grade 0). The microscopic lesion score in the liver tissue from diabetes groups was 4 with severe distortion of the structure and large perivascular fibrosis areas compared to the control group. Exercise and IGF-I treated groups were 2, which was an indication of moderate fibrosis thickening of liver tissue with a refined structure of liver tissue compared to the DIA group. Moreover, combination therapy did not attenuate perivascular fibrosis induced by diabetes on liver tissue compared to the DIA + EX and DIA + IGF-I groups.

**Fig. 7:**
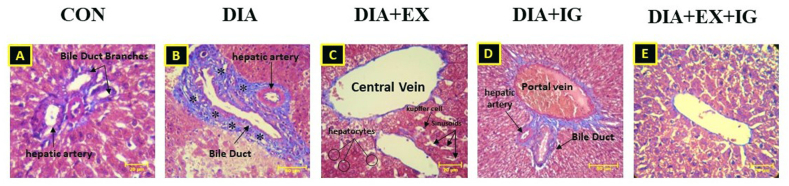
(A–E): Photomicrographs of liver tissue staining by Masson Trichrome. The star indicates fibrosis.

## 4 Discussion

The main findings of this study were as follows: diabetic rats showed increased lipid levels and liver enzymes in their plasma. This undesirable effect of diabetes was alleviated after treatment, either alone or in combination with IGF and exercise. Furthermore, diabetic rats exhibited elevated ER stress with increased UPR components in their livers, which were reduced after treatment with IGF and exercise. The study also observed increased fat accumulation in the livers of diabetic rats, which was influenced by changes in the protein expression of genes involved in lipid metabolism (FXR, SREBP-1, FAS). These conditions improved after treatment with IGF and exercise, demonstrating a potent synergistic effect of IGF and exercise in the simultaneous treatment of NAFLD and ER stress signaling pathways.

Nonalcoholic fatty liver disease (NAFLD) is a progressive complaint that can lead to steatosis, cirrhosis, fibrosis, impaired liver function, and liver failure [26]. Today, it is clear that NAFLD has developed as a common chronic disease in diabetic patients. Despite the prevalence of NAFLD in diabetic patients, the precise molecular mechanisms of diabetes remain poorly understood [26]. Some studies revealed that increased translation of SREBP-1C [27], FAS genes [28], and defective FXR [29] protein regulation in the diabetes condition facilitates the occurrence of fatty liver in diabetes.

In line with these reports, our results showed increased protein levels of FAS and SREBP-1C. This led to decreased protein levels of FXR in the diabetic group, possibly mediating fat accumulation in the liver.

To find the reasons for the protein expression changes in the liver of diabetic models, we paid attention to the molecular changes of the endoplasmic reticulum homeostasis due to a strong association between lipid metabolism and endoplasmic reticulum. Owing to the high ability of hepatocytes for protein synthesis, it has been shown that the unfolded protein response (UPR)/ER stress signaling pathways significantly mediate pathological changes in numerous liver diseases [30,31].

In particular, ER stress and UPR-related pathways have been reported to play an effective role in the development of fat accumulation, steatosis, and progression to nonalcoholic steatohepatitis by changing the expression of molecules involved in lipid metabolism [32]. Although many research studies offer some reasons for ER stress response-induced steatosis, it remains uncertain whether stressed UPR contributes to hepatic steatosis.

Elevated ER stress markers such as GRP78/BiP, ATF4, ATF6, and CHOP have been shown in the liver of diabetic rats [33]. Expression levels of GRP78/BiP as an essential chaperone in the appropriate protein folding increased under impaired protein folding and cell stress [34]. In the present study, elevated GRP78 levels in the liver of diabetic animals indicated activation of ER stress, which may have been involved in fat accumulation.

During ER stress stimulation, the PERK signaling pathway is activated, which induces the activation of ATF4 by eIF2α [35]. ATF4 is one of the main influences that initiate CHOP apoptosis in cells [35]. Furthermore, the crosstalk between the PERK signaling pathway and the molecules involved in lipid metabolism is obvious [36]. Results showed that elevated levels of PERK occurred in the diabetic groups induced overexpression of caspase 8 and 12 and the induction of apoptosis mediated by the CHOP signaling pathway [36]. Wang et al. in 2018 reported that Asiatic acid inhibits NAFLD and decreases expression levels of FAS and SREBP-1 by reducing the extent of ERS [37]. Our result also showed that activation of the PERK signaling pathway, caspase 8 and 12, and NAFLD markers was significantly inhibited by treatment with IGF-I and exercise.

Moreover, the second pathway stimulated by ER stress is the IRE1α signaling pathway. The IRE1α pathway is highly related to hepatic lipid metabolism that can lead to the activation of some enzymes that are involved in TG biosynthesis [38]. In this study, it was shown that increased expression of IRE1α parallel to increased TG was induced in diabetic groups. On the other hand, the results indicated a decrease in IRE1α expression levels in the IGF-I and exercise-treated groups.

The third pathway induced in ER stress is the ATF6 branch which plays a crucial role in stress-induced lipid accumulation [39]. Exercise has been found to reduce ER stress proteins GRP78 and ATF6 in NAFLD [39]. We found that IGF-I treatment and exercise partially prevented the level of ATF6 expression in diabetic groups. Treatment with IGF-I and exercise suppressed activation of ER stress which was revealed by a reduction in GRP78, IRE-1A, ATF6, and CHOP. By altering the expression of essential enzymes involved in lipid synthesis, ER stress, and UPR pathways play a crucial role in lipid metabolism. Patients with NAFLD showed activation of several UPR components [32]. Also, it has been shown that CHOP activation induces hepatic lipid accumulation through PPARγ expression during ER stress [40].

In this study, in parallel with increased levels of CHOP in the liver of diabetic rats, the protein levels of FAS and SREBP-1C increased, and protein levels of FXR decreased, mediating lipid metabolism perturbation. In line with our work, it is reported that increased ER stress in the hepatocytes increased the expression of SREBP-1c, which activated fatty acid synthesis [41]. Kammoun, H. showed that in the liver, the forced expression of GRP78 results in a reduction of hepatic steatosis and improvement of glucose homeostasis by preventing SREBP-1c activation [42]. In this regard, our result determined that in diabetes conditions, there was an increased level of CHOP in parallel with increased GRP78 that mediated apoptosis by caspase 8, 12, which means high levels of GRP78 could not improve lipid metabolism in diabetic animals.

It is also shown that high levels of PERK-eIF increased lipid accumulation by activating SREBP-1c and SREBP-2 [41].

Our result showed that increased ER stress is accompanied by decreased FXR expression, which may mediate steatosis in the liver of the diabetic animal. It is reported that inhibition of ER stress in old mice by Tauroursodeoxycholic acid treatment increased FXR expression with reduced TG in the liver, suggestive of the improving effect of FXR in ER stress-induced lipotoxicity [20]. According to the results of our study, it may be that activation of ER stress in diabetic animals has been involved in liver fat accumulation. As a result of exercise and IGF-I treatment, the activation of ER stress was somewhat reduced. Apoptosis is induced by ER stress during the maladaptive phase using CHOP, an ER stress transcription factor. Three classic UPR pathways can activate CHOP, including PERK, IRE1-XBP1, and ATF6 [43]. Our result demonstrated elevated levels of cleaved caspase 8 and 12 in the liver of diabetic rats as a result of elevated CHOP and that treatment with IGF-I and exercise extinguished this condition.

Insulin-like growth factor-1 (IGF-I) is a peptide hormone structurally related to insulin that plays a crucial role in regulating cell survival, proliferation, differentiation, and metabolism [44]. It is clear that IGF-I insufficiency is common in type 1 diabetes, and as a result of this decrease, the sensitivity of cells to insulin decreases [44].

NAFLD and NASH models have demonstrated antifibrotic properties of IGF-I in rodent models. However, the exact molecular mechanism that is used by IGF-I to improve NAFLD has yet to be made clear [45].

Furthermore, it was shown that IGF-I protects cells from the apoptosis consequences of the maladaptive phase of ER stress and enhances cell adaptive stress response by introducing posttranslational modifications of target proteins [46]. Our results also showed a protective effect for IGF-I on liver steatosis, as IGF-I improved protein expression of FXR, SRECBP-1, and FAS that are involved in NAFLD features. It seems that IGF-I, due to its functional similarity to insulin, reduces liver fat accumulation in diabetic rats by suppressing various ER stress markers such as CHOP, IRE-1A, and ATF6 ER stress.

Today, it is clear that regular exercise is an influential factor in preventing liver diseases in healthy people and people with metabolic diseases [47]. Hajighasem et al. in 2018, showed that exercise increases FXR gene expression and decreases fat accumulation in the liver of healthy rats [47]. On the other hand, according to Chengji et al.'s studies, treadmill exercise reduces ER stress and related signaling pathways in the heart tissue of diabetic rats [48]. Our result also showed that mild treadmill exercise decreased ER stress in the liver of diabetic rats, decreased protein expression of SRECBP-1 and FAS, and increased protein expression of FXR. Furthermore, another outcome in the present study was the synergistic decreasing effect of IGF-I and exercise on ERS levels and fat accumulation in the liver.

A limitation of this research is the need for confirmation of the overall conclusion about the effects of IGF-I and exercise on ER-stress markers by using ER-stress pathway inhibitors or activators. In addition, using other ER-stress markers besides CHOP can help detect changes caused by post-translational modifications.

## 5 Conclusion

Finally, the results discovered that IGF-I and exercise decrease fat accumulation in the liver of diabetic animal models by suppressing ER stress signaling pathways. Furthermore, although we did not assess the exact molecular signaling of IGF-I and exercise on ER stress markers in this research, it is partially understood that IGF-I and exercise synergistically reduced ER stress by moderating the PERK, ATF6, IRE-1A, and CHOP signaling pathways. IGF-I and exercise, alone or together, can control maladaptive ER stress situations by inducting an adaptive ER stress status and stimulating protein folding properly. The results also suggest that the use of IGF-I and exercise in the clinical treatment strategy may be valuable in the treatment of NAFLD in diabetic people as a novel healing plan.

## Ethics declarations

This study was reviewed and approved by Animal Ethic Committee of the Urmia University of Medical Sciences, with the approval number: [IR.UMSU.REC.1399.099].

## Data availability statement

The data used and analyzed in the current study are available from the corresponding author upon a reasonable request.

## CRediT authorship contribution statement

**Shadi Mohammadpour-Asl:** Writing – original draft, Project administration, Formal analysis, Data curation. **Behrad Roshan-Milani:** Software, Data curation. **Shiva Roshan-Milani:** Writing – review & editing, Conceptualization. **Ehsan Saboory:** Writing – review & editing. **Bijan Ghobadian:** Writing – review & editing. **Leila Chodari:** Writing – review & editing, Supervision, Project administration, Methodology, Investigation, Funding acquisition.

## Declaration of competing interest

The authors declare that they have no known competing financial interests or personal relationships that could have appeared to influence the work reported in this paper.

## Figures and Tables

**figs1:**
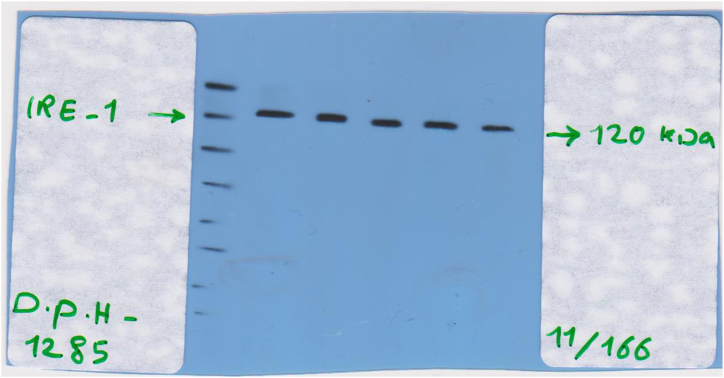


**figs2:**
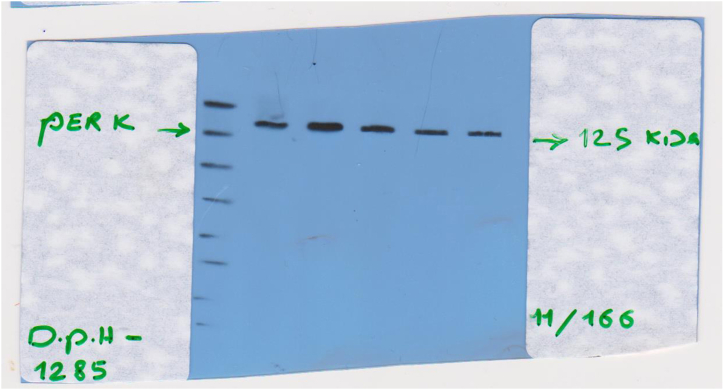


**figs3:**
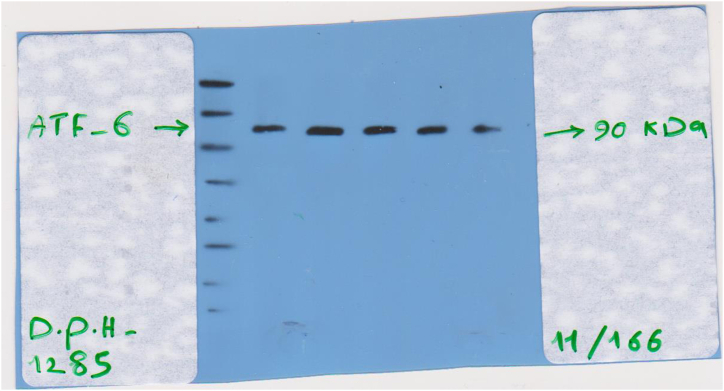


**figs4:**
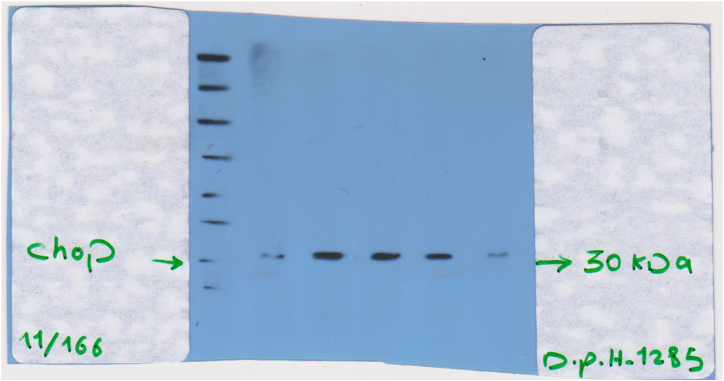


**figs5:**
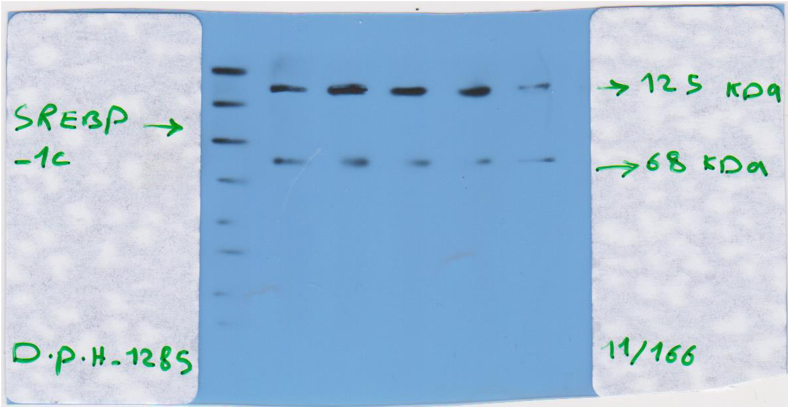


**figs6:**
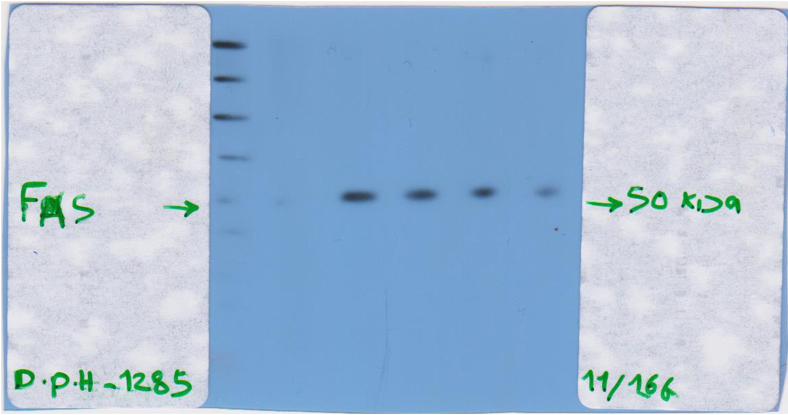


**figs7:**
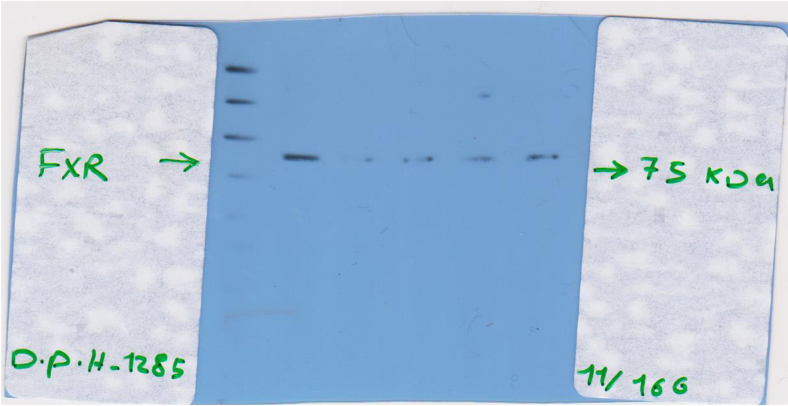


**figs8:**
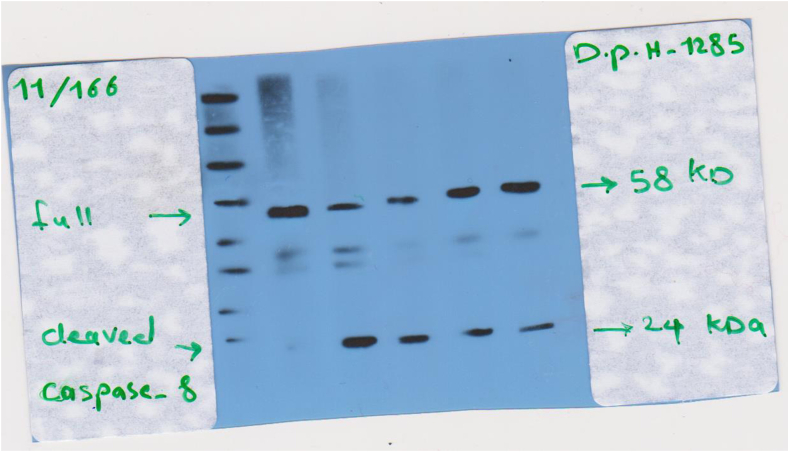


**figs9:**